# A Case Study of Calcium Carbonate Crystallization during Reverse Osmosis Water Desalination in Presence of Novel Fluorescent-Tagged Antiscalants

**DOI:** 10.3390/membranes12020194

**Published:** 2022-02-06

**Authors:** Konstantin Popov, Maxim Oshchepkov, Alexei Pervov, Vladimir Golovesov, Anastasia Ryabova, Maria Trukhina, Sergey Tkachenko

**Affiliations:** 1 JSC “Fine Chemicals R&D Centre”, Krasnobogatyrskaya, Str. 42, b 1, 107258 Moscow, Russia; maxim.os@mail.ru (M.O.); golovesov.vova@mail.ru (V.G.); truheniy-mv@yandex.ru (M.T.); s.tkach.8@gmail.com (S.T.); 2 Department of Chemistry and Technology of Biomedical Pharmaceuticals, Mendeleev University of Chemical Technology, 125047 Moscow, Russia; 3 Department of Water Supply and Sewage, Moscow State University of Civil Engineering, Yaroslavskoe Shosse, 26, 129337 Moscow, Russia; ale-pervov@yandex.ru; 4 Prokhorov General Physics Institute of the Russian Academy of Sciences, Vavilov Str., 38, 119991 Moscow, Russia; nastya.ryabova@gmail.com

**Keywords:** reverse osmosis, membrane fouling, calcium carbonate scaling, fluorescent-tagged polyacrylate, fluorescent-tagged bisphosphonate, fluorescence, scale inhibition

## Abstract

Calcium carbonate scaling in reverse osmosis (RO) desalination process is studied in the presence of two novel fluorescent-tagged scale inhibitors 1,8-naphthalimide-tagged polyacrylate (PAA-F1) and 1-hydroxy-7-(6-methoxy-1,3-dioxo-1*H*-benzo[de]isoquinolin-2(*3H*)-yl)heptane-1,1-diyl-bis(phosphonic acid) (HEDP-F) by fluorescent microscopy (FM) and scanning electron microscopy (SEM). Both antiscalants diminished the mean size of calcite crystals relative to the blank experiment. The behavior and localization of HEDP-F and PAA-F1 during calcite scale formation on membrane surface was found to be significantly different from the distribution in similar RO experiments with gypsum, reported earlier. In the former case, both antiscalants are concentrated exactly on the surface of calcium carbonate crystals, while in the latter one they form their own phases (Ca-HEDP-F and Ca-PAA-F1) and are not detected on gypsum scale. The difference is interpreted in terms of interplay between background calcium concentration and sparingly soluble calcium salts’ solubility. HEDP-F reveals slightly higher efficiency than PAA-F1 against calcite scale formation, while PAA-F exhibits a higher ability to change calcite morphology. It is demonstrated that there is a lack of correlation between antiscaling efficacy and ability of antiscalant to change calcium carbonate morphology in a particular case study. An application of fluorescent-tagged antiscalants in RO experiments provides a unique possibility to track the scale inhibitor molecules’ localization during calcite scale formation. Fluorescent-tagged antiscalants are presumed to become a very powerful tool in membrane scaling inhibition studies.

## 1. Introduction

Reverse osmosis (RO) has become a powerful technology for the purification of sea, brackish and waste water [1,2,3,4]. RO is currently the most energy-efficient technology for desalination, with an energy cost of about 1.8 kWh/m^3^, which is much lower than that of other technologies [3]. However, one of the major limitations in efficient RO application is the surface membrane fouling [4,5]. It is a complex phenomenon and could be a combination of colloidal, organic, inorganic and biofouling [4].

Membrane fouling could significantly reduce productivity and permeate quality. The former leads to increasing operation costs due to increased energy demands, additional pretreatment, foulants’ removal and membrane cleaning and maintenance, as well as reduction in membrane lifetime [3]. Inorganic fouling or scaling is the formation of mineral deposits on the membrane surface as the feed water becomes supersaturated by inorganic salts. The relative content of dissolved salts is increased four to ten times, depending on the operating recovery and rejection efficiencies [4] during high pressure membrane operations. As a result, the concentration of salts may exceed their solubility limit and the salts may crystallize onto membrane surfaces. The most common scales are represented by calcium carbonate, calcium sulfate and silica [2,4,6,7]. Scale formation has always been a serious limitation in designing and operating RO systems since scaling causes flux decline, membrane degradation, loss of production and elevated operating costs.

Scaling was reported to occur by two crystallization pathways, surface crystallization and bulk crystallization [4]. Scale deposition in membrane systems is a combination of these two extreme mechanisms and is affected by membrane morphology and process conditions. Surface crystallization occurs due to the lateral growth of the scale deposit on the membrane surface, resulting in flux decline and surface blockage. Bulk crystallization arises when crystal particles are formed in the bulk phase through homogeneous crystallization and may deposit on membrane surfaces as sediments/particles to form a cake layer that leads to flux decline. In addition, supersaturated scale forming conditions lead to scale growth and agglomeration [4].

To mitigate the risk of membrane scaling, many methods, including feed water pretreatment, the optimization of operational processes, the development of novel membrane materials and the addition of antiscalants, have been developed in accordance with the characteristics of RO operating systems [1,5,8]. An application of antiscalants provides retardation of scale formation, and increases the concentration coefficient of concentrate. It is one of the most commonly applied methods for inhibiting scaling in the RO process, especially in the situation that a high-water recovery is set to reduce concentrate volume but cause an enhanced scaling potential, due to its high efficiency, low cost and easy operation [8].

Among scale inhibitors, polycarboxylates (polyacrylates, polyaspartates, etc.) and phosphonates are found to be highly efficient [2,4,9]. However, in spite of the availability of a good number and wide variety of effective antiscalants, continuous research is being conducted to improve the existing ones [2,9,10,11]. Recent reviews on scale formation control in RO technologies [2,4,8,9,12] mention hypothetical mechanisms of inhibition: antiscalant molecules adsorb to the active growth sites on the crystal surface of sparingly soluble inorganic salt, formed either in the bulk feeding solution or at the membrane surface. This sorption delays the onset crystallization (nucleation phase of crystallization) or retards the growth of mineral salt crystals (growth phase of crystallization) [4,12].

However, in spite of numerous relevant studies, the lack of understanding of the complicated physicochemical interactions involved among dominant scale-forming substances, antiscalants, membranes and other coexisting substances in solutions still exists [8,11,13,14]. One of the promising possibilities to eliminate some gaps in antiscalant mechanisms understanding is a direct [13,14] or indirect [11] visualization of antiscalant. Indeed, our recent experiments with gypsum scale formation in a laboratory RO facility in the presence of fluorescent-tagged bisphosphonate antiscalant 1-hydroxy-7-(6-methoxy-1,3-dioxo-1*H*-benzo[de]isoquinolin-2(*3H*)-yl)heptane-1,1-diyl-bis(phosphonic acid), HEDP-F (H_4_hedp-F) [13] and of fluorescent 1,8-naphthalimide-tagged polyacrylate (PAA-F1) [14] revealed a paradoxical effect: an antiscalant does not show any interaction with gypsum at all, but provides nevertheless retardation of corresponding deposit formation.

**Figure 1:**
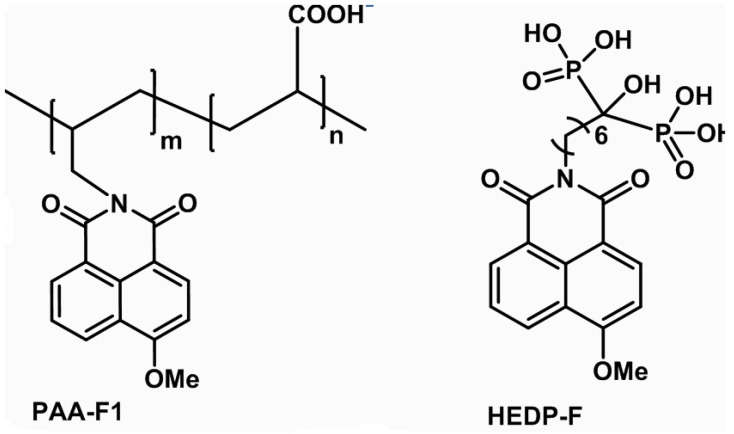
PAA-F1 and HEDP-F molecular structures.

Thus, it was of particular interest to track both fluorescent scale inhibitors during calcite deposition on RO membranes in a model experiment. The present study is focused therefore on the scale inhibitor visualization after RO treatment of a model water sample with high calcium carbonate content in the presence of two different fluorescent antiscalants—PAA-F1 and HEDP-F (Figure 1). One of them represents polycarboxylates (PAA-F1), while another one represents organophosphonic acids (HEDP-F). The major objective of the present work was to model the naturally occurring calcium carbonate supersaturation process as the feeding solution steadily gets saturated, and to ascertain the scale inhibitor location. As far as we know, this is a first full-scale report on antiscalant’s visualization during calcite fouling onto membrane surface in a RO experiment.

## 2. Materials and Methods

### 2.1. Reagents

Antiscalant PAA-F1 (Figure 1) was synthesized by our group, as described elsewhere [15]. It has the mean molecular mass 4000 Da with c.a. 1 mass % of fluorescent 1,8-naphthalimide moiety. This corresponds randomly to c.a. 0.2 fluorescent fragments per one molecule of polyacrylate. HEDP-F (molecular mass 502.1 Da), (Figure 1), was synthesized by our group as described in [16]. For model scaling solutions, reagent grade CaCl_2_·2H_2_O and Na_2_CO_3_ were used in crystalline form and were separately dissolved in distilled water (conductivity 2 μS/cm) to prepare stock solutions.

### 2.2. Calcium Carbonate Feed Solutions Preparation

Following this study objectives, we had to start experimental runs with feeding aqueous solution, undersaturated relative to calcium carbonate. At the same time, at the end of each run the retentate has to become supersaturated, and capable of generating a sufficient amount of calcite crystals for scanning electron microscope (SEM) detection, and for confocal luminescent microscope analysis (FM) in both antiscalnt-injected and blank experiments. Thus, the dosage of antiscalant should not be too high in order not to suppress scaling completely.

The solubility of calcite in water at 25 °C and zero ionic strength (*I*) corresponds to 0.058 mmol·dm^−3^ [17]. However, at pH below 10 a higher concentration of calcium and carbonate ions in aqueous phase becomes possible due to HCO_3_^−^ anions formation:

CaCO_3 calcite_ ⇆ [CaCO_3_ ]_solution_ + H^+^ ⇆ [CaHCO_3_]^+^ ⇆Ca^2+^ + HCO_3_^−^ (1)

**Figure 2:**
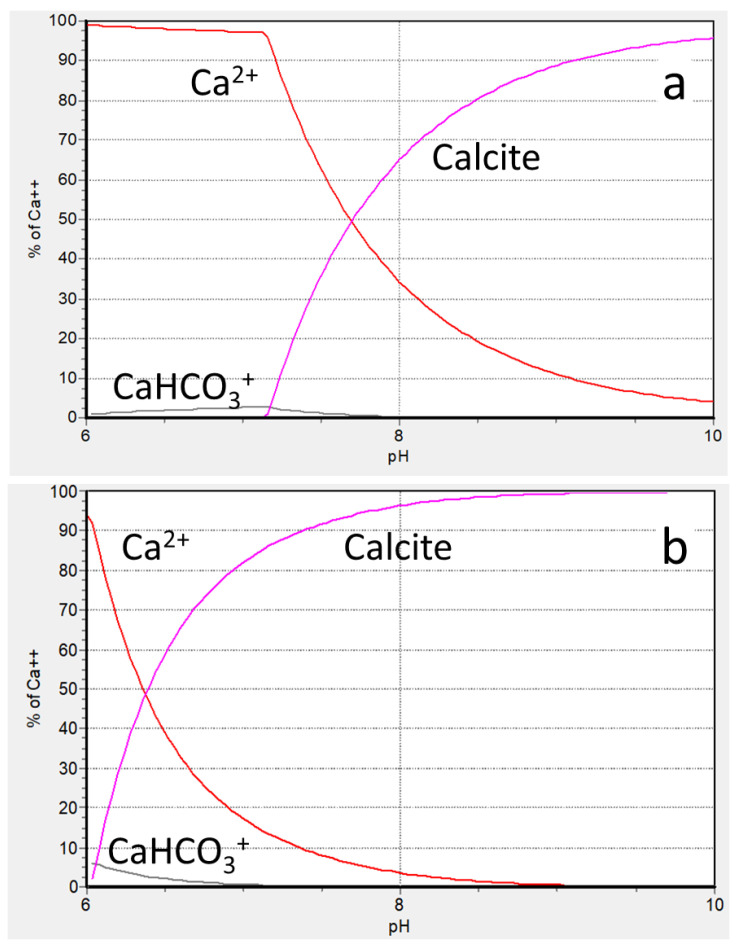
Chemical speciations for 0.25 (**a**) and 1.2 (**b**) mmol·dm^−3^ CaCO_3_ as a function of pH at 25 °C and *I* = 0 mmol·dm^−3^.

In order to find optimal experimental conditions, the chemical speciations for calcium have been performed, based on chemical equilibria data [17,18,19,20], operating SPECIES software [21], (Table 1, Table 2 and Figure 2).

The data, presented in Table 2 and in Figure 2a, demonstrate that the initial 2.5 mmol·dm^−3^ calcium carbonate solution has no solid phase below pH 7.1, while above this boundary calcite should appear in significant amounts, reaching 95 mol % at pH 10. For more concentrated calcium carbonate solutions (15 mmol·dm^−3^) the boundary of heterogeneity is shifted to more acidic solutions (Figure 2b). Thus, if 2.5 mmol·dm^−3^ calcium carbonate solution is prepared at pH 7.1, it would be a homogeneous liquid phase with 1% confidence. A six-fold saturation of this solution in a RO facility at the same pH should lead to the formation of solid phase (calcite), which would accumulate no less than 79 mol % of total calcium, present in retentate (Figure 2b). Following these speciation diagrams the stock CaCl_2_·2H_2_O and Na_2_CO_3_ solutions have been prepared in such concentrations, and with such pH, that being mixed in 1:1 volume ratio they would give a 2.5 mmol·dm^−3^ calcium carbonate solution at pH 7.9, that is above the saturation level: 1.5 mmol calcite (60%) has to be formed at this pH value. Notably, the supersaturation (S) of initial 2.5 mmol·dm^−3^ CaCO_3_ solution is ranging from 2.5 (pH 7.9) to 5.0 (pH 8.4) according to diagram at Figure 2a. Here S is denoted as [Ca_init_]/[Ca_equil_], where [Ca_init_] indicates initial calcium concentration, and [Ca_equil_] indicates equilibrium calcium concentration according to the diagram at Figure 2a. However, due to the slow kinetics of solid phase formation, the initial feed solution remains homogeneous after CaCl_2_·2H_2_O and Na_2_CO_3_ solutions get mixed. Meanwhile, the higher initial pH provides a higher yield of calcite crystals at the end of an experiment.

Each stock solution was filtered (200 nm; Hydrophilic PTFE Millipore Millex-LG membrane) before mixing to remove dust particles. An antiscalant was added to the feed solution before use to achieve 5 mg·dm^−3^ dosage. This corresponds to c.a. 0.07 mmol·dm^−3^ PAA-F1 concentration (−CH_2_CH<^COOH^ fragments) and to 0.01 mmol·dm^−3^ HEDP-F concentration.

### 2.3. Reverse Osmosis Membrane System

**Figure 3:**
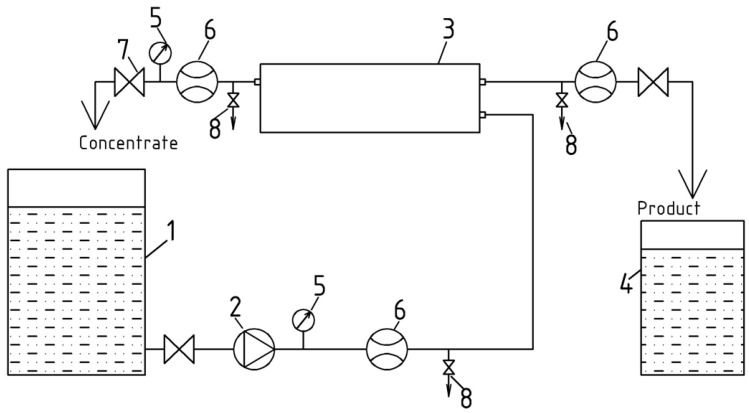
Schematic diagram of laboratory RO unit for membrane scaling tests: 1—feed water tank; 2—pump; 3—spiral wound membrane module; 4—permeate tank; 5—pressure gauge; 6—water flow meter; 7—concentrate flow adjusting valve; 8—sampler.

Calcite scaling experiments were carried out using an automatically controlled laboratory-scale cross-flow RO spiral wound module RE 1812 tailored with thin film composite BLN membrane (Figure 3). The test unit was operated in circulation mode whereby concentrate after membrane module was returned back to feed water tank. The feed water was added to feed water tank 1. The volume of tank 1 was 5 L. Feed water from tank 1 was supplied by small gear pump 2 to membrane module 3. In all the experiments capacities were used comprising commercially available spiral-wound filter elements (CSM RE1812-80 GPD) made of polyamide and manufactured by CSM (Korea) operated at constant feed flow rate of 72 ± 0.2 dm^3^/h, permeate flow rate of 6.0-6.3 dm^3^/h, constant temperature of 25.0 ± 1 °C; constant pressure 7 ± 0.2 bar in concentration mode. The area of membrane surface was 0.5 m^2^.

Stabilized salt rejection, at a constant pressure of 7 bar, solution temperature of 25 °C, and a pH value of 6.5–7.0 is reported to be 96.5–97.0 % for a 200 mg·dm^−3^ NaCl solution (manufacturer’s data). Each run was performed with a virgin membrane sample. Flux recovery was 80 to 83%.

### 2.4. Calcium Carbonate Scaling Experiments

Individual calcite scaling experiments were performed with a virgin sample of a pre-soaked membrane: each membrane contacted with distilled water overnight (12–14 h) to allow membrane permeability to stabilize. Experiments were run with a single superficial cross-flow velocity and were terminated after reaching concentration coefficient K = 6. The cross-flow velocity varied from 3.0 to 3.6 cm/s. This value adequately fits the range of cross-flow velocities encountered in spiral-wound RO/NF. The retentate was periodically sampled and tested for calcium content (EDTA titration) and pH. Pressure and retentate cross-flow rates were monitored through digital sensors. The permeate volume was continuously recorded. The temperature was almost constant (varying by less than 1 °C) during each experiment at a level of 25 °C.

At the end of each experimental cycle, scaled membrane samples were carefully extracted from the autopsied membrane element and submerged into an ultrapure water bath for approximately 2 s to prevent further crystallization from evaporation of residual scaling solution. Membrane samples were then air dried for at least 48 h and afterwards cut into ten equally sized 4 cm × 10 cm pieces. These pieces were stored in desiccators for at least 24 h. Then the fragments were sent for analysis by scanning electron microscopy (SEM) and by fluorescent microscopy (FM).

### 2.5. Fluorescent Microscopy Measurements

Confocal microscopy measurements have been run with laser scanning confocal microscope LSM-710-NLO (Carl Zeiss Microscopy, Jena, Germany), 20× Plan-Apochromat objective (NA = 0.8). The membrane samples were placed onto a Petri dish with a glass bottom 0.16 mm thick. The fluorescence was recorded in the wavelength range of 470–600 nm, when excited by laser radiation with a wavelength of 458 nm. It should be noted that 1,8-naphthalimide-based antiscalants provide fluorescence in the blue spectral region. However, the fluorescent channel can be assigned any pseudo-color in the digital image when acquired by laser scanning microscopy. Most often the fluorescent channel, if it is a single one, is assigned the green pseudo-color because the dynamic range of human color perception for green is much wider than for blue. Thus, in the present work the original blue emission is changed for an artificial green pseudo-color. In some cases, an overlay of the fluorescent images with distribution of PAA-F1 and HEDP-F (green pseudo-color in images) and transmitted light image (grey color) was obtained.

### 2.6. SEM Crystal Characterization

The membrane pieces with precipitated solids, after being triply rinsed with deionized water and air dried at 20–25 °C, were characterized by scanning electron microscopy (SEM, Hitachi TM-3030, Tokyo, Japan). The sample examinations by SEM were done at 15 kV accelerating voltage in a Charge-Up Reduction Mode with crystal phase located on Conducting Double-Sided Tape and 4.1 mm working distance.

## 3. Results and Discussion

### 3.1. Variation of Calcium Concentration in Retentate

**Figure 4:**
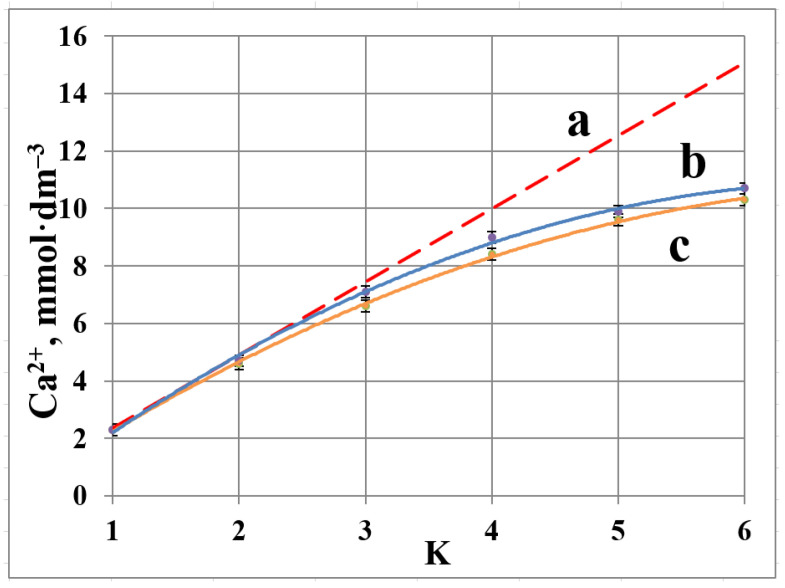
Variation of total calcium content in retentate + membrane (**a**); of calcium concentration in retentate in presence of HEDP-F (**b**); and in retentate in a blank run (**c**).

The data on calcium concentration in a blank experiment reveal a notable deviation from linearity (dashed line) for concentration coefficient K = 2 (Figure 4). This indicates accumulation of Ca^2+^ ions in solid phase. The difference between the total calcium content (retentate + membrane; dashed line) and its concentration in the liquid phase (Figure 4c) increases steadily as K values are changing from 3 to 6. Thus, almost 30% of calcium gets deposited as calcite at the end of experiment, while for K < 2 all calcium species are found in a liquid phase. At the same time, pH values remain constant within the range 7.9 ÷ 8.5 (Table 3).

Notably, calcite deposition on membrane surface (30%) appears to be less than the expected equilibrium value (95%). This difference is attributed partly to the uncompleted equilibrium state of the system and partly to the loss of some carbonate ions in the form of CO_2_ (some bubbles have been detected in permeate).

**Figure 5:**
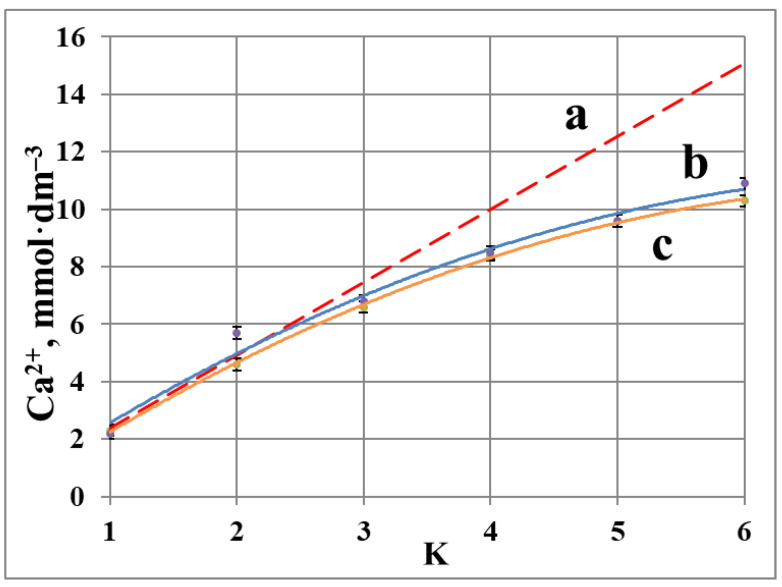
Variation of total calcium content in retentate + membrane (**a**); of calcium concentration in retentate in the presence of PAA-F1 (**b**); and in retentate in a blank run (**c**).

An incorporation of antiscalant diminishes scale deposition for K = 2 and 3, indicating a definite scale inhibition phenomenon. However, at K = 6 a 5 mg·dm^−3^ dosage fails to retard scaling, and the corresponding “b” and “c” curves demonstrate similar values for both HEDP-F and PAA-F1, Figure 4 and Figure 5. At the same time, HEDP-F reveals a better antiscaling performance relative to PAA-F1 at K = 2 and K = 3. This is in reasonable agreement with static tests, run for the non-fluorescent analogues of HEDP-F and PAA-F1: HEDP and PAA [22,23,24].

### 3.2. SEM and FM Analysis of Membrane Surface

**Figure 6:**
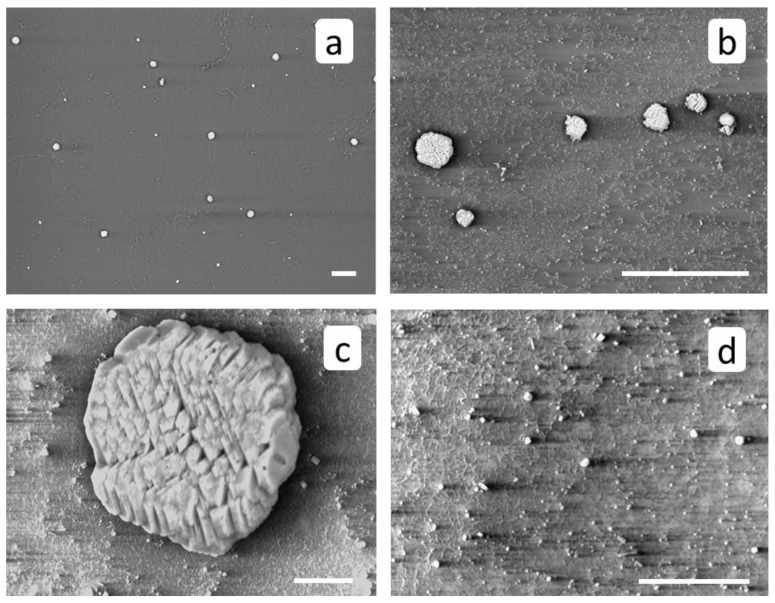
SEM images of RO membrane surface after a blank run. Scale marker corresponds to 100 μm (**a**,**b**) or to 10 μm (**c**,**d**).

SEM images of RO membrane surface after a blank run (Figure 6) demonstrate a small number of isolated crystals and crystal clusters, typical for calcite [4,22]. Along with large aggregates with sizes around 20 to 30 μm, Figure 6c, there is also a fine fraction (sizes 500 nm and less) (Figure 6d). On the whole, the calcium carbonate phase covers much less than 1% of membrane surface.

**Figure 7:**
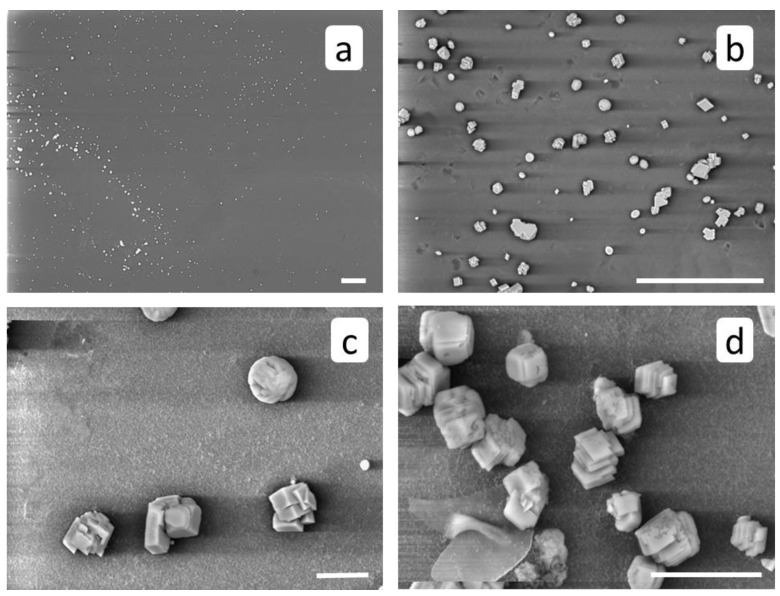
SEM images of membrane surface after RO treatment in the presence of HEDP-F. Scale marker corresponds to 100 μm (**a**,**b**) or to 10 μm (**c**,**d**).

After RO experiment with HEDP-F, calcium carbonate crystals reveal less uniform distribution on the membrane’s surface (Figure 7a), and a smaller mean size relative to the blank experiment: c.a. 10 μm, Figure 7b–d. However, some particle aggregates with sizes around 50 μm are also detected. The morphology of crystals does not seem to change significantly.

**Figure 8:**
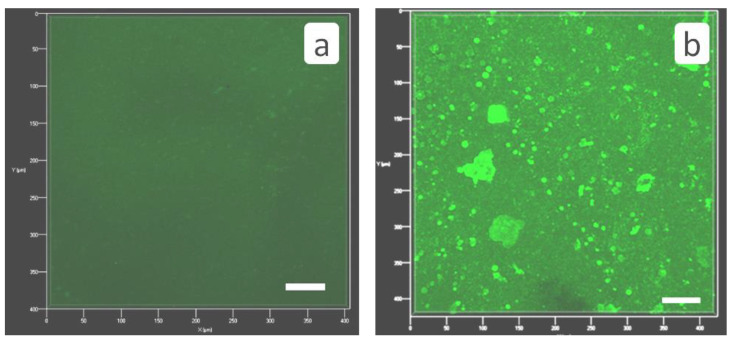
3D fluorescent images of virgin untreated membrane (**a**); and of membrane surface after RO treatment in the presence of HEDP-F (**b**). HEDP-F fluorescence—green pseudo-color, Scale marker corresponds to 50 μm.

**Figure 9:**
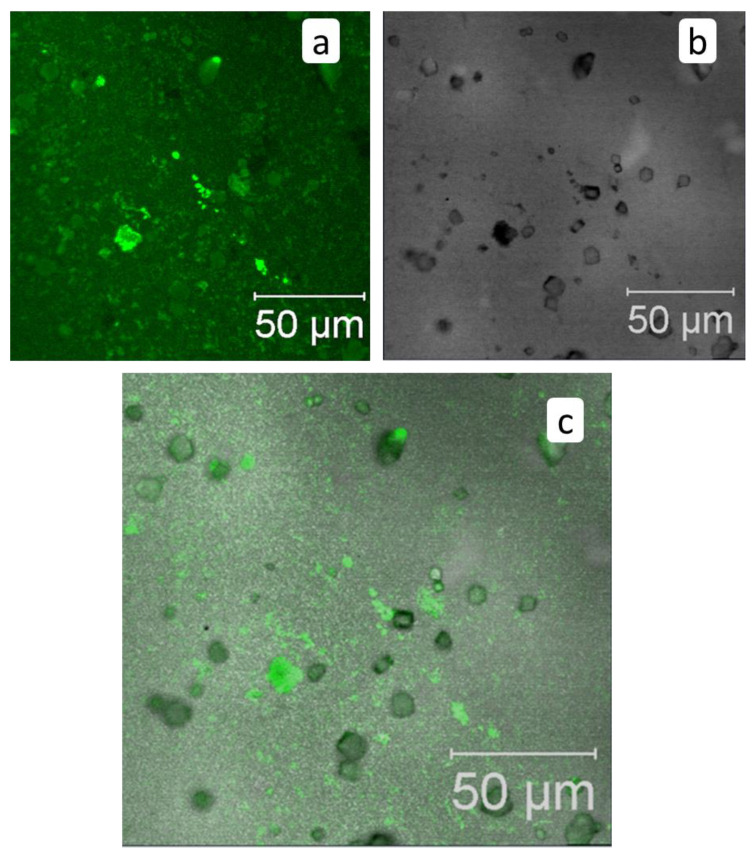
Fluorescent images of membrane surface after RO treatment in presence of HEDP-F: **a**—HEDP-F fluorescence—green pseudo-color, **b**—transmitted light image (grey color), **c**—imposition of **a**,**b** channels.

Virgin membrane surface exhibits a weak fluorescence itself (Figure 8a), but this fluorescence does not mask antiscalant (Figure 8b). Fluorescent images of membrane surface after RO treatment in the presence of HEDP-F demonstrate, that antiscalant is concentrated exactly on the surface of calcium carbonate crystals, Figure 8b and Figure 9. Notably, an “inside” location of HEDP-F is normally visible as a shining crystal center with a dark crystal surface [13,16]. Although, one can’t exclude an antiscalant presence in the form of traces (which are beyond the detection limits) inside the crystals, but this effect is definitely negligible relative to dominating fluorescence exactly on the calcite surface. A dominant location of HEDP-F on calcite surface is very much different from its distribution, observed by us earlier in a similar RO experiment with gypsum [13]. In the case of CaSO_4_·2H_2_O scale formation HEDP-F is completely accumulated in its own solid phase (Ca-HEDP-F), but it was not detected on gypsum crystals at all. At the same time, HEDP-F distribution on CaCO_3_ surface is not uniform: some crystals are covered by an antiscalant completely, some have accumulated HEDP-F only at the edges, and some reveal either traces of fluorescent marker or even the lack of its presence (Figure 9c). Meanwhile, Figure 9c indicates also some small green spots on the membrane surface, which can be attributed either to Ca-HEDP-F solids or to HEDP-F, unevenly adsorbed directly to the membrane surface.

Tentatively, the differences in HEDP-F location between RO experiments with CaSO_4_·2H_2_O and CaCO_3_ scaling are associated with initial (Ca^2+^) concentration, and with Ca/HEDP-F mole ratio. In gypsum scaling experiments the calcium content was adjusted to 15 mmol·dm^−3^, and 0.014 mmol·dm^−3^ HEDP-F dosage was used [13]. In the recent study, all experiments started with [Ca^2+^] = 2.5 mmol·dm^−3^, while HEDP-F dosage was almost the same as in [13]: 0.01 mmol·dm^−3^. Thus, in a gypsum scaling experiment most of antiscalant was accumulated in Ca-HEDP-F phase long before retentate got supersaturated relative to CaSO_4_·2H_2_O. This fact was well documented by FM measurements of liquid phase [13]. We can’t rule out the possibility of Ca-HEDP-F phase formation in the present study. These are normally observed as spherical green species [14,16]. However, a confident assignment of green spots on membrane surface (Figure 9c) to solid Ca-HEDP-F particles is hardly possible, as far as visually they look similar to fine particles of calcite. On the other hand, a significant part of 25 mg of HEDP-F, present in 5 L of stock solution, is adsorbed on macro-crystals of CaCO_3_ as a surface layer. Thus, a very little amount, if any, is left for a separate Ca-HEDP-F phase formation.

At the same time, the relative rates of CaSO_4_·2H_2_O, CaCO_3_ and Ca-HEDP-F formation at equal supersaturation may also become responsible for the observed differences. Besides, a different sorption ability of HEDP-F on CaCO_3_ and CaSO_4_·2H_2_O surfaces at pH 7 ÷ 8 may also give some contribution to these differences.

At first glance, the data presented in Figure 4, Figure 7, Figure 8 and Figure 9, prove the conventional mechanism of inhibition: antiscalant molecules adsorb at the CaCO_3_ crystal surface active growth sites, and retard crystal growth and aggregation [12]. Indeed, the mean size of calcite crystals, deposited in presence of HEDP-F, is smaller than that one in a blank experiment. Besides, FM indicates a nonuniform localization of HEDP-F on some calcite particles with higher accumulation of antiscalant exactly on the crystal edges and faces. On the other hand, the sizes of antiscalant-covered and uncovered calcite crystals (Figure 9c) are almost the same. This fact indicates, that the sorption of HEDP-F on the surface of already formed CaCO_3_ plays the secondary role (if any) in retardation of scale deposition. Thus, a nonconventional mechanism, reported by us earlier for gypsum [13,14,19], can also be applied in this particular case. It was proposed that antiscalant molecules block not the nuclei of sparingly soluble salt and the surface of its macrocrystals, but the surface of solid nano/micro impurities, that are always present in any aqueous solution, even after its filtration with 200 nm pore membranes [25,26,27]. These nano/micro impurities (nanodust) act as promoters of heterogeneous nucleation of sparingly soluble salts. Their blockage by antiscalant molecules diminishes the number of crystallization centers, available for calcite nucleation, and causes retardation of initial (nucleation) steps of scale formation. As a result, calcite solution in presence of antiscalant becomes more supersaturated than the blank one. This in turn leads to the formation of smaller sized crystals.

**Figure 10:**
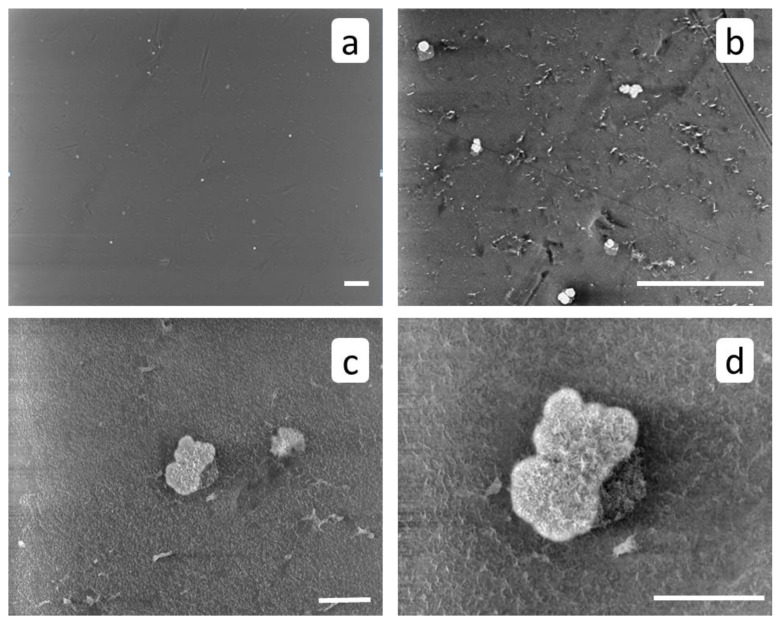
SEM images of membrane surface after RO treatment in presence of PAA-F1; Scale marker corresponds to 100 μm (**a**,**b**); or to 10 μm (**c**,**d**).

**Figure 11:**
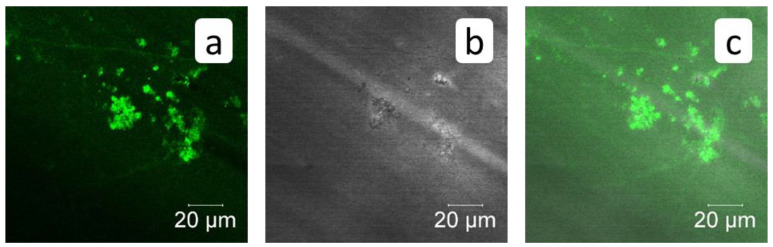
Fluorescent images of membrane surface after RO treatment in the presence of PAA-F1. (**a**)—PAA-F1 fluorescence—green pseudo-color; (**b**)—transmitted light image (grey color); (**c**)—imposition of (**a**,**b**) channels.

The RO experiment with PAA-F1 (Figure 10 and Figure 11) demonstrates the same effect as with HEDP-F. The mean size of deposited crystals (Figure 10a) is smaller than of those, formed in a blank experiment (Figure 6a). Antiscalant is also accumulated mostly on calcite crystals (Figure 11a). Meanwhile, in the case of calcite PAA-F1, it also demonstrates behavior different to gypsum scale formation [14]. In RO experiments with gypsum, PAA-F1, like HEDP-F, does not interact with CaSO_4_·2H_2_O crystals, but forms individual solid phase Ca-PAA-F1.

At the same time, unlike HEDP-F, polyacrylate definitely causes a well-expressed crystal morphology change of calcium carbonate (Figure 10d). Although such effects have been registered earlier both in our experiments [28], and in experiments of other research groups [29,30], this observation should still be noted. The less efficient antiscalant PAA-F1 causes morphology change of calcite, while the more efficient HEDP-F does not. This result casts doubt on the validity of the conventional statement, that the crystal habit change by molecules of antiscalant is an approval of its scale inhibition activity [12]. Current observation gives an additional indication that a correlation between antiscalant’s efficacy and its ability to change scale crystal habit is generally missing.

A comparison of HEDP-F and PAA-F1 distribution onto calcite surface also reveals some differences. PAA-F1 covers the CaCO_3_ surface completely and rather uniformly, while HEDP-F demonstrates a nonuniform location: some crystals of calcium carbonate appear to be completely uncovered, some are partly covered, while some are covered completely. The present study indicates, along with latest reports [27,31], that fluorescent-tagged scale inhibitors are capable to provide valuable information on the nature of scale inhibition.

## 4. Conclusions

It is found that the behavior and localization of HEDP-F and PAA-F1 during calcite scale formation on membrane surface is very different from their distribution in similar RO experiments with gypsum. In the former case both antiscalants are concentrated exactly on the surface of calcium carbonate crystals, while in the latter case they form their own phases: Ca-HEDP-F and Ca-PAA-F1. The difference is interpreted in terms of interplay between background calcium concentration and sparingly soluble calcium salts solubility.

HEDP-F and PAA-F1 reveal different distribution onto calcite surface: PAA-F1 covers the CaCO_3_ surface completely and rather uniformly, while HEDP-F demonstrates uneven location.

The lack of correlation between the efficacy of the antiscalant and its ability to change the morphology of the calcium carbonate in the particular case study has been demonstrated.

The utility of chemical speciations for the preliminary choice of calcium carbonate solutions (pH, initial concentration) is shown.

An application of fluorescent-tagged antiscalants in RO experiments provides a unique possibility to track the scale inhibitor molecules’ location during calcite scale formation.

## Figures and Tables

**Table 1:** Equilibrium constants (log*K*), used for equilibrium speciations at 25 °C and *I* = 0.

Equilibrium	log*K* (-log*K*_sol_)	Reference
Ca^2+^+ CO_3_^2-^ ⇆ CaCO_3 calcite_	8.485	[18]
Ca^2+^+ CO_3_^2-^ ⇆ [CaCO_3_ ]_solution_	3.22	[17]
H^+^+ CO_3_^2−^ ⇆ HCO_3_^−^	10.34	[19]
H^+^+ HCO_3_^−^ ⇆ H_2_CO_3_*where [H_2_CO_3_*] = [CO_2_(aq)] + [H_2_CO_3_]	6.36	[19]
Ca^2+^+ HCO_3_^−^ ⇆ CaHCO_3_^+^	1.14	[20]

**Table 2:** Calcium species’ distribution as a function of pH at 25 °C and *I* = 0 mol dm^−3^.

pH	Calcium Species, % (mol)			
	Ca^2+^	CaCO_3 solution_	[CaHCO_3_]^+^	CaCO_3 calcite_
	Initial solution [Ca^2+^] = 2.5 mmol·dm^−3^; [HCO_3_^−^] = 2.5 mmol·dm^−3^			
7.00	97.2	0.15	2.65	0.0
7.12	97.0	0.20	2.77	0.0
7.16	95.9	0.22	2.73	1.19
7.20	91.0	0.22	2.49	6.29
7.40	70.6	0.22	1.57	27.62
7.80	43.4	0.22	0.63	55.71
8.00	34.3	0.22	0.40	65.08
9.00	11.0	0.22	0.04	88.78
	The same solution, concentrated (6-fold)			
6.00	94.4	0.03	5.60	0
6.08	87.7	0.04	5.48	6.78
6.20	70.1	0.04	4.16	25.73
6.80	26.2	0.04	1.04	72.69
7.00	19.8	0.04	0.66	79.52
8.00	5.7	0.04	0.07	94.18

**Table 3:** Variation of calcium concentration and pH in retentate.

K	Blank Experiment		In Presence of PAA-F1, 5 mg·dm^−3^		In Presence of HEDP-F, 5 mg·dm^−3^	
	pH	Ca^2+^, mmol·dm^−3^	pH	Ca^2+^, mmol·dm^−3^	pH	Ca^2+^, mmol·dm^−3^
1	7.9	2.4 ± 0.1	8.2	2.5 ± 0.1	8.4	2.5 ± 0.1
2	8.5	4.6 ± 0.1	8.6	5.6 ± 0.1	8.3	5.5 ± 0.1
3	8.4	6.6 ± 0.1	8.5	7.2 ± 0.2	8.3	7.4 ± 0.1
4	8.3	8.4 ± 0.1	8.5	8.5 ± 0.1	8.3	9.0 ± 0.1
5	8.1	9.6 ± 0.1	8.5	9.5 ± 0.1	8.1	9.9 ± 0.1
6	8.0	10.3 ± 0.1	8.4	10.9 ± 0.1	8.0	10.6 ± 0.1
